# Survey on sanitary practices and knowledge about infectious diseases among equine owners in the State of Rio Grande do Norte, Brazil

**DOI:** 10.29374/2527-2179.bjvm003323

**Published:** 2023-11-27

**Authors:** Paulo Roberto Medeiros, Liliany Silva Figueiredo, Ubiratan Pereira de Melo, Amanda Louíse Bittencourt Mariz, Emilson Lima de Brito, Ingrid Raquel dos Santos Araújo, Allyson Lincoln Da Costa Silva, Mariana Henrique da Silveira Costa, Cintia Ferreira, Daniel Barbosa Assis, Camilla Raylly Miguel da Silva, Aldemir Lopes de Souza, Magna Pereira da Silva de Souza

**Affiliations:** 1 1- Undergraduate in Veterinary Medicine, Grupo de estudos e pesquisa em medicina equina (GEPMEq), Centro Universitário Mauricio de Nassau, Natal, RN, Brazil.; 2 2- Veterinarian, D.Sc. GEPMEq, Centro Universitário Mauricio de Nassau, Natal, RN, Brazil.

**Keywords:** deworming, equine, infectious disease, sanitary management, desparasitação, equinos, doenças infecciosas, manejo sanitário

## Abstract

As the primary decision-maker about the health, nutrition, and well-being of their horses, owners' knowledge of correct management practices and clinical changes can potentially affect the immediate health of their horses, in addition to having an impact on the prevention of disease spread in the herd. The adoption of management practices to prevent the introduction and spread of pathogens depends on various factors, including demographics, awareness of the problem, perceived responsibility, previously held beliefs, and sociocultural norms. This study aimed to evaluate the health management practices and the level of knowledge about infectious diseases of equine owners in the state of Rio Grande do Norte, Brazil. A cross-sectional study was conducted by distributing a questionnaire to horse owners in Rio Grande do Norte, Brazil. The participants included horse owners irrespective of the duration of ownership, experience, or sports practiced. In addition to the hygiene and management of animals, the questionnaire addressed topics related to the facilities where the horses were kept. Three hundred and two horse owners distributed in 60 of the 167 municipalities in the state of Rio Grande do Norte participated in this study. Among the interviewed owners, 63.90% (193/302) answered that they do not quarantine animals introduced into the herd or those visiting the property. Regarding the deworming program, 95.03% (287/302) of the owners regularly dewormed their animals. However, more than half of the participants sought veterinary guidance (54.30%). Investing in educational programs and increasing the awareness of equine owners in the state of Rio Grande do Norte about the main infectious diseases in horses is essential toward improving sanitary management and the general health of animals. Informed owners play an essential role in creating safer environments for their horses, thereby contributing to the sustainability of the equine industry.

## Resumo

Como principal tomador de decisão sobre a saúde, nutrição e bem-estar de seus cavalos, o conhecimento dos proprietários sobre práticas corretas de manejo e mudanças clínicas pode potencialmente afetar a saúde imediata de seus cavalos, além de ter um impacto na prevenção de propagação das doenças no rebanho. A adoção de práticas de manejo para prevenir a introdução e disseminação de patógenos depende de uma variedade de fatores, incluindo dados demográficos, consciência do problema, responsabilidade percebida, crenças anteriores e normas socioculturais. Este trabalho teve como objetivo avaliar as práticas de manejo da saúde e o nível de conhecimento sobre doenças infecciosas de proprietários de equinos no estado do Rio Grande do Norte, Brasil Brasil. As amostras incluíram proprietários de cavalos, independentemente da duração da posse, experiência ou esporte praticado. O questionário abordou temas relacionados às instalações onde os cavalos eram mantidos, além do manejo higiênico-sanitário dos animais. Participaram do estudo 302 proprietários de cavalos distribuídos em 60 dos 167 municípios do estado do Rio Grande do Norte. Dentre os proprietários entrevistados, 63,90% (193/302) responderam que não colocam em quarentena os animais introduzidos no rebanho ou que estão visitando a propriedade. Em relação ao programa de desverminação, 95,03% (287/302) proprietários administravam vermifugação regularmente aos seus animais. No entanto, pouco mais da metade procura orientação veterinária (54,30%). Investir em programas de educação e conscientização dos proprietários de equinos do estado do Rio Grande do Norte sobre as principais doenças infecciosas em equinos é fundamental para melhorar o manejo sanitário e a saúde geral dos animais. Proprietários informados desempenham um papel essencial na criação de ambientes mais seguros para os cavalos, contribuindo para a sustentabilidade da indústria equina como um todo.

## Introduction

Horse owners can expect to find changes in their animal's health condition over their lifespan, with large-scale surveys showing that approximately one-third of horses suffer from at least one health condition each year. As the primary decision-makers regarding the health, nutrition, and well-being of their horses, owners' knowledge of correct management practices and clinical changes can potentially affect the immediate health of their horses, in addition to preventing the spread of the disease in the herd (Golding et al., 2023).

As a general contingency measure to prevent the spread of infectious diseases, some of which are zoonotic in nature, horse owners should implement disease prevention measures as part of their routine management. Several recommended prevention measures are similar for endemic and exotic diseases, including vaccination, quarantine of newly arrived horses, and good hygiene (Spence et al., 2019).

The adoption of management practices to prevent the introduction and spread of pathogens depends on various factors, including demographics, awareness of the problem, perceived responsibility, previously held beliefs, and sociocultural norms. Individuals make judgements about the “risk” of health risks and the subsequent level of prevention they should implement to counteract those risks. Horse owners' attitudes and perceptions of disease risk factors may influence their adoption of disease prevention measures (Spence et al., 2019).

Assessment of horse owners' knowledge about clinical signs of infectious and parasitic diseases, how the disease is spread, and whether vaccines are available to control or limit disease spread are important factors to consider, as owner compliance with preventive and control measures would be required in the event of a disease outbreak. This information could guide horse owners’ education strategies regarding the risk of diseases and how to recognize clinical signs in affected horses and could assist early recognition of diseases, particularly in situations of heightened disease risk (Chapman et al., 2018).

Questionnaires are commonly used in veterinary research and can make important contributions to knowledge (Christley, 2016). Although survey studies using questionnaires on the level of knowledge of animal owners regarding various diseases are common practices in several countries (Bowden et al., 2020; Golding et al., 2023), this tool is rarely used in Brazil (Arruda et al., 2022; Babá et al., 2013; Costa et al., 2022).

This study aimed to evaluate the health management practices and level of knowledge about infectious diseases among equine owners in Rio Grande do Norte, Brazil.

## Material and methods

This cross-sectional study was conducted by distributing a questionnaire to horse owners in Rio Grande do Norte, Brazil. The participants were horse owners, irrespective of the duration of ownership, experience, or sports practiced. Owners who agreed to participate in the study were informed about the research purpose as well as assured of the confidentiality of their identity, that of their animals, and of any other collected data.

After obtaining oral and/or written consent, the authors individually administered the questionnaires within a convenient sampling framework. The owners answered the questionnaire independently; that is, the authors did not help the owners, except when the owner was illiterate or had a physical disability. In this study, one of the authors helped the owner complete the questionnaire without influencing the responses. The questionnaire was sent to the owners with no specified deadlines.

In addition to the hygiene and sanitation management of these facilities, the questionnaire addressed topics related to the facilities where the horses were kept. Detailed questions were asked about the condition of the pens and the regularly performed cleaning and disinfection procedures. In addition, the survey included specific questions about the quarantine period for newly arrived animals as well as its duration. Another aspect addressed was vaccination and deworming protocols.

Data were collected from December 2022 to March 2023 and stored in Microsoft Excel® spreadsheets for later statistical analysis performed using the free Epi Info 7.2 software (Centers for Disease Control and Prevention, 2023).

## Results

Three hundred and two horse owners distributed across 60 of the 167 municipalities in the state of Rio Grande do Norte participated in this study. Of these, 83.77% (253/302) were male, while 16.23% were female.

According to the data depicted in Table 1, 58.28% (176/302) of the interviewees kept their horses in stalls and paddocks, whereas 33.45% (101/302) remained exclusively in stalls. Regardless of the stall being the only facility where horses were kept or used in conjunction with paddocks, 95.30% (264/277) of the respondents responded that the stall bed was composed exclusively of sand. Regarding the use of a drainage system in the bays, only 37.18% (103/277) of the interviewees answered that it existed.

**Table 1:** Type and basic management of facilities utilized for the maintenance of horses in Rio Grande do Norte state, Brazil.

**Variable**	**Total**	**%**
**In which environment the animal is kept?**		
Stall	101	33.45
Paddock	25	8.27
Stall and Paddock	176	58.28
***Total***	302	100
**If the horse stays all day or part of the day in the stall, what type of floor is there?**		
Sand	264	95.30
Wood shavings	6	2.16
Other material	7	2.54
***Total***	277	100
**If the animal remains in a stall, does it have drainage?**		
Yes	103	37.18
No	174	62.82
***Total***	277	100
**How often is the stall bed changed?**		
Monthly	55	19.86
Every two months	65	23.46
Every four months	78	28.16
Semiannually	60	21.66
Annually	19	6.86
***Total***	277	100
**How is the handling of waste from the stall, especially feces?**		
Feces are removed once a day	69	24.90
Feces are removed twice a day	207	74.73
only when necessary	1	0.37
***Total***	277	100
**After removing the waste from the stall, how are handled?**		
Feces undergo processing (biodigestion, composting etc.) before being used as biofertilizers	8	2.89
Feces are accumulated outdoors and sold to fertilizer companies	21	7.58
Feces are discarded in an environment far from the facilities	67	24.19
The feces are deposited in the weeds/paddock in order to serve as natural fertilizer	70	25.27
Faeces are deposited close to the facilities	111	40.07
***Total***	277	100

Table 2 presents data related to general sanitary management practices. Among the interviewed owners, 63.90% (193/302) answered that they do not quarantine animals introduced to the herd or that are visiting the property. Among the 36.10% (109/302) of the owners who performed quarantine, the period of quarantine ranged from 1 to 10 days. In relation to carrying out a sanitary vacuum or cleaning the stalls when changing horses, 54.30% (164/302) answered that no action was carried out other than only changing horses. On the other hand, 45.70% (138/302) responded that they carried out a sanitary emptying before housing a horse in a previously occupied stall. Regarding immunization practices, 95.03% (287/302) of respondents vaccinated their animals. Among those who adopted vaccination as a health management practice, 71.43% (205/302) responded that vaccination was performed annually.

**Table 2:** Sanitary management practices adopted by owners horses in the state of Rio Grande do Norte.

**Variable**	**Total**	**%**
**Do you quarantine new animals acquired or that are entering/visiting the property?**		
Yes	109	36.10
No	193	63.90
***Total***	302	100
**If the previous answer was YES, what is the quarantine period adopted**		
1-2 days	15	13.76
3-6 days	38	34.86
7-10 days	21	19.26
Over 10 days	35	32.12
***Total***	109	100
**When there is a change of stall between horses or the horse is placed in a stall previously occupied by another horse, what management is adopted in relation to the stall?**		
Only sanitary vacuum is carried out	138	45.70
Nothing is accomplished. The horse is just placed in the stall	164	54.30
***Total***	302	100
**It carries out vaccination of animals?**		
Yes	287	95.03
No	15	4.97
***Total***	302	100
**What is the interval between vaccinations?**		
Annually	205	71.43
Every two years	8	2.79
Sporadically	27	9.41
Semiannually	47	16.37
***Total***	287	100
**You seek veterinary advice for guidance on the vaccination program?**		
Yes	195	64.57
No	107	35.43
***Total***	302	100
***In the event of infectious or contagious infectious disease, which of the alternatives best describes the conduct adopted in relation to the isolation of affected animals?***		
It depends on the diagnosis and guidelines of the Veterinarian	150	49.67
It isolates initially, but when the horse presents a small improvement, it returns again to the conviviality of the others.	53	17.55
No isolation measures are adopted	99	32.78
***Total***	302	100

**Figure 1:**
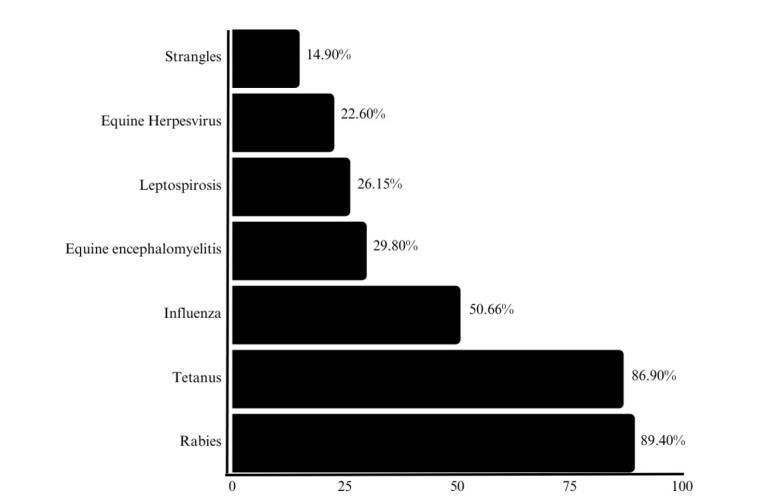
Main diseases for which horses are immunized by owners in the state of Rio Grande do Norte, Brazil.

**Figure 2:**
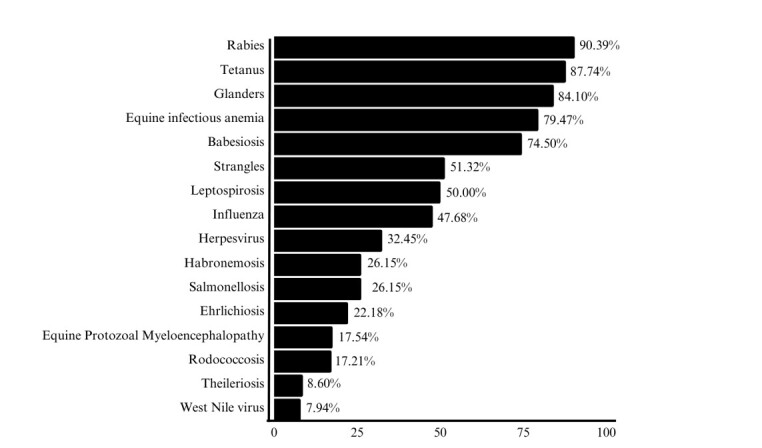
Main infectious diseases known to horse owners in the state of Rio Grande do Norte, Brazil.

Regarding the diseases included in the immunization program, 89.40% of interviewees answered that they immunized their horses against rabies, 86.90% against tetanus, and 50.66% against equine influenza. Other cited diseases were encephalomyelitis, leptospirosis, herpes viruses, and strangles (Figure 1). Figure 2 shows the main infectious diseases identified by interviewees.

Regarding the deworming program, 95.03% (287/302) of the owners regularly dewormed their animals (Table 3). However, more than half of the participants sought veterinary guidance (54.30%). The interval between the deworming administrations varied from 30 to 180 days. According to the answers obtained from the questionnaire, the main reason for deworming was to prevent diseases associated with nematodes. Among the anthelmintic drugs available on the market, the most commonly used was ivermectin (81.13%), followed by doramectin, moxidectin, and trichlorfon.

**Table 3:** Management practices adopted by horse owners to control intestinal parasites in the state of Rio Grande do Norte.

**Variable**	**Total**	**%**
**Animals are dewormed regularly?**		
Yes	287	95.03
No	15	4.97
***Total***	302	100
**Do you seek medical veterinary guidance regarding the correct use of dewormers?**		
Yes	164	54.30
No	138	45.70
***Total***	302	100
**What is the basis for the initiative to carry out deworming?**		
Identification of clinical signs of diseases associated with worms	41	13.57
Veterinary guidance	93	30.80
Prevention of diseases	168	55.63
***Total***	302	100
**How often is deworming performed?**		
Monthly	32	10.60
Every two months	69	22.84
Every three months	69	22.84
Every four months	62	20.53
Semiannually	70	23.19
***Total***	302	100
**Do you usually rotate deworming drugs?**		
Yes	196	64.90
No	106	35.10
***Total***	302	100
**Which dewormers have you used in the last 12 months?**		
Ivermectin	245	81.13
Doramectin	25	8.28
Moxidectin	22	7.28
Trichlorfon	10	3.31
***Total***	302	100
**Faecal egg count test (FECT) is performed to determine the need for deworming?**		
Yes	29	9.60
No	107	35.43
I didn't know there was such a technique	166	54.97
	302	100

**Figure 3:**
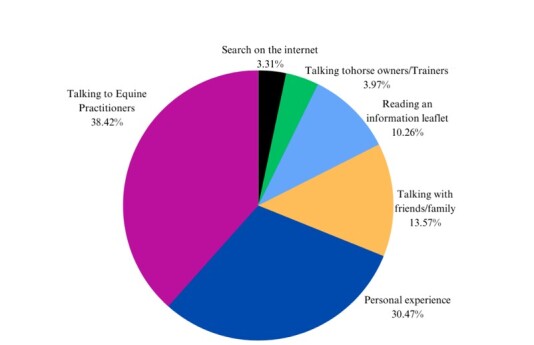
Sources for the search for information among horse owners in the state of Rio Grande do Norte, Brazil.

Figure 3 presents the main sources of information on disease management among the interviewed horse owners. The main sources of knowledge cited were conversations with veterinarians (38.42%) and personal experiences gained over time (30.47%).

## Discussion

The data obtained through this study make it possible to show that the majority of the interviewed horse owners adopted a semi-intensive breeding regime, and a smaller proportion adopted intensive and extensive regimes. These results are similar to those obtained by Melo and Ferreira (2023) in a retrospective study on dental alterations in vaquejada horses, where athletic horses were kept most of the day in stalls with restricted access to paddocks during the night. A similar observation was made by Dias et al. (2013) in a cross-sectional study that showed that 53.6% of the horses used in vaquejada were bred in a semi-intensive regime with access to paddocks and remained in the stall for over 11 hours a day.

Some owners doubt the importance of dedicating care to the type of surface on which the animal is housed in the stalls. In addition to respiratory problems (Calciolari et al., 2019; Melo et al., 2007), due to the strong presence of ammonia, especially when the urine is not well drained due to the absence of a drainage system in the stall, an erroneous posture can predispose horses to numerous diseases, including hoof diseases (Melo et al., 2009). A large number of materials are used as bedding in stables, with different costs and material availability depending on the owner’s region. Sand is widely used in the northeastern region of Brazil, and in some places, it is the only efficient way to make beds for horses.

Infectious diseases pose a constant threat to the health and welfare of horses. Several diseases have assumed greater importance. Further, as performance of horse populations and equine activities have increased, and there are new owners who do not understand the implications of equine infectious disease outbreaks on their animals. Vaccination and preventive management procedures are critical for disease prevention; however, they need to be more widely adopted, complied with, and promulgated (Roberts, 2014). In this context, quarantine of new animals introduced into the herd or those that are in transit on the property is vital for preventing the spread of diseases among horses. The data obtained from this survey showed that 63.90% (193/302) of the interviewed owners had not adopted quarantine as a preventive measure to control infectious diseases.

The fact that there should be a separate facility used for any horses that have left the venue for equestrian events and not just newly introduced or sick horses is worth noting (Roberts, 2014). However, quarantining animals returning from competitions is not routine in the study region. The main equestrian sport in this region is vaquejada. In some events there is an agglomeration of animals of different origins and under inadequate health conditions due to lack of sanitary control, which constitute risk factors for the transmission of several infectious diseases, as suggested by Lage et al. (2007) and Taques et al. (2021). Recently, Melo and Ferreira (2022a) described five simultaneous incidences of bacterial pneumonia associated with *E. coli* in athletic horses. History revealed that three animals that had recently left the property for competition were reintroduced into the facility without being quarantined, spreading the disease to three more animals.

Biosecurity represents an important aspect of the veterinarians’ responsibilities on farms, stables, barns, and at shows and events where horses mix from multiple locations. Technicians, owners, and caregivers must understand the implications, share responsibilities, and implement specific management procedures to prevent disease spread (Roberts, 2014). The data obtained from this survey showed that 49.67% (150/302) adopted biosecurity measures after clinical diagnosis and following veterinarian guidance. The exchange of information between veterinarians and horse owners during an infectious disease outbreak plays an important role in the effectiveness of biosecurity measures adopted in the herd. Specialist veterinarians are an important source of information on infection control for managers, as they enable the assessment of disease risk and control options (Schemann et al., 2012).

Planning for an infectious disease outbreak can also be enhanced by understanding the biosecurity practices adopted at individual farms or training facilities, as these measures can be an effective barrier to prevent the transmission of infectious diseases when horses are transferred between farms or returned from competitions. Although many farm owners and managers are aware of the need for biosecurity, only a few implement the practices that could prevent the spread of infectious diseases (Rosanowski et al., 2013).

The main infectious diseases (Figure 2) known by the interviewed owners were rabies (90.39%), tetanus (87.74%), glanders (84.10%), equine infectious anemia (79.47%), babesiosis (74.50%), strangles (51.32%), and leptospirosis (50.00%). The main diseases mentioned by owners are those commonly reported in the national literature (Coelho et al., 2022; De Faria et al., 2022; Ferreira et al., 2021; Melo & Ferreira, 2022b). However, other diseases for which clinical cases have already been reported in Brazil or antibodies circulating in different regions, such as equine herpes virus (Estima-Silva et al., 2019), equine encephalitis (Nunes et al., 2021; Diniz et al., 2022), and West Nile virus, are little known or totally unknown to owners.

Education and awareness among owners, veterinarians, and professionals involved in equine management are essential for the early detection of possible diseases and the implementation of appropriate preventive measures. By recognizing the disease and its early symptoms, owners can seek veterinary assistance as quickly as possible, which increases the chances of effective treatment and containment of the problem before it becomes a full-scale outbreak. Additionally, the owners’ active participation in control and prevention strategies is crucial to the success of these measures. They must follow biosecurity guidelines, implement adequate vaccination programs, and adopt management measures to reduce the risk of disease transmission among animals. Recognition of the importance of infectious diseases in horses by the owners to understand the relevance of their role in the early identification and notification of these diseases is essential.

When equine owners are well informed about the importance of early identification of infectious diseases, prompt reporting of suspected cases, and the adoption of proper biosecurity practices, they directly contribute to the prevention of outbreaks that can affect both animal and human health.

In recent decades, nematodes have been recognized as an important cause of disease and performance loss in horses. Horse owners have long used interval dosing, which involves administering anthelmintic drugs at fixed intervals throughout the year to all horses in a herd (Losinno et al., 2018; Melo et al., 2011). Based on the prepatent period and climatic conditions, treatment is recommended every 2 to 4 months (Losinno et al., 2018). This recommendation seems to have been adopted by the majority of the owners in this study, and as documented by Lignon et al. (2021), this approach remains mainly due to the ease of application, acquisition, and cost-effectiveness for breeders.

The findings of this study suggest that carrying out coprological examinations (fecal egg counting) was an uncommon practice among the interviewed owners. Of the interviewed owners, only 9.60% (29/302) carried out the test periodically, whereas 90.40% (273/302) never carried out the test or were unaware of its existence. The results obtained in relation to the use of cropological examination as a tool for decision-making and monitoring the effectiveness of the anti-nematode program were lower than those reported previously (Bolwell et al., 2015; Losinno et al., 2018). Surveys in other countries have reported signs of a changing approach towards deworming, with the increasing use of fecal egg counts (FEC) monitoring and reduced treatment intensity (Nielsen et al., 2018).

The widespread occurrence of anthelmintic resistance in equine parasites worldwide has led to recommendations for FEC-based parasite programs to reduce treatment intensity, thereby delaying the further development of resistance as much as possible (Nielsen et al., 2018). Strategies for parasite control have undergone major changes in recent years, replacing the traditional approaches with those based on surveillance (diagnosis) and selective therapies. Selective therapy recognizes a small percentage of the equine population carrying a high parasite load and a much larger percentage with very low or zero FEC. The concept that parasite populations are unequally distributed among a group of hosts is the basic premise of selective therapy. Although there is no uniform way of practicing it, in general, this approach is based on regular monitoring of equine populations through FEC in all animals; however, only those who have exceeded a predetermined threshold receive treatment (Losinno et al., 2018).

According to the results of this study, the administration of anthelmintic drugs at fixed times was almost exclusively the only measure aimed at controlling the parasites in the studied population. This finding is worrisome, as 54.30% (164/302) of owners responded that they sought veterinary advice for deworming. This finding may indicate a failure in owner-veterinarian communication, mainly because 54.97% (166/302) were unaware of the new recommendations for the management of nematodes.

Like other grazing animals, horses are exposed to intestinal parasites present in the soil, which have the potential to cause gastrointestinal illnesses. Although the use of anthelmintics has reduced the prevalence of the pathogenic parasite *Strongylus vulgaris* to low levels, it has also led to the development of anthelmintic resistance in *Cyathostominae* and *Parascaris* spp. This highlights the need for a shift towards more sustainable control approaches that limit or prevent the development of resistance (Osterman-Lind et al., 2022).

To limit the use of anthelmintic drugs, alternatives, such as proper fecal and pasture management methods, should be employed to minimize horse exposure to intestinal parasites (Osterman-Lind et al., 2022). The present study demonstrated that 89.53% of the owners discarded the feces removed from the stalls in the paddock or in the equine living environment, without any other management being carried out. This can result in contamination of the pastures by nematodes and a vicious cycle of infection, which can culminate in the indiscriminate use of anthelmintics in parasitic resistance. In a recent study, Osterman-Lind et al. (2022) demonstrated that by maintaining clean pastures by removing feces twice a week, a previously parasite-free pasture can remain almost completely free of infective larvae for a five-week grazing period or longer up to three months.

The quarantine of newly arrived animals or visitors is also considered important to reduce the contamination of stalls and pastures with populations of resistant parasites. However, only 36.10% of the owners interviewed adopted this management strategy. These findings are similar to those of studies conducted in other countries where there was a low level of quarantine (Fritzen et al., 2010; Traversa et al., 2009). According to Elghryani et al. (2019), it is difficult to compare owners’ attitudes towards the implementation of quarantine because the adoption of this conduct is influenced by various factors such as the number of animals, the farm size, and the concomitant manifestation of other infectious diseases.

Although 54.30% of the interviewed owners sought guidance from equine practitioners on the proper use of anthelmintic drugs, the data relating to other methods that can be used in the proper management of intestinal parasites illustrates that more efforts must be made by veterinarians to disseminate current methods of intestinal parasite control among owners.

The administration of anthelmintics to animals is an important component of programs aimed at controlling and preventing parasitic infections. Among the available compounds, four distinct chemical groups are most commonly used: benzimidazoles (e.g., albendazole, fenbendazole, and oxibendazole), pyrimidines (e.g., pyrantel pamoate), and macrocyclic lactones (e.g., ivermectin, moxidectin, abamectin, and doramectin). In recent decades, the indiscriminate use of these drugs has contributed to the emergence of anthelmintic-resistant nematode populations, especially those belonging to the subfamily Cyathostominae, commonly known as cyathostomines. This seriously threatens health, well-being, and equine production in several areas across the world (Barbosa et al., 2018). The data from this study showed that the interviewed parents almost exclusively used anthelmintics from the macrocyclic lactone group, notably ivermectin.

Although more than half of the interviewees (64.90%) answered that they rotated between the anthelmintic drugs used in the deworming programs, this does not translate to what is seen in the clinical routine, as in several situations, the owner only changes the trademark, but the pharmacological basis remains the same. This finding highlighted that the almost exclusive and indiscriminate use of macrocyclic lactones can result in parasite resistance.

Obtaining knowledge about diseases is essential for adequately and efficiently addressing them. Several sources contribute to the expansion of this knowledge, each with its own advantages and limitations. According to the results of this survey, owners seek information or attribute their acquired knowledge about diseases and management to various sources, notably conversations with veterinarians (38.42%) and personal experiences acquired over the years (30.47%). Personal experience can be a valuable resource as it allows us to quickly understand the clinical signs and impacts of diseases. However, this knowledge is limited to specific and common cases in that property or region and does not cover the diversity of the clinical manifestations of a disease. Consulting technical information is essential for supporting knowledge with up-to-date information from studies, but its use may be restricted to literate owners, making it impossible for the uneducated owners to access the information. Conversations and the exchange of information with equine veterinarians with the expertise to interpret and apply acquired knowledge properly are crucial for a solid and reliable level of knowledge.

The results of this survey are similar to those by Bowden et al. (2020), who documented the level of knowledge of owners about equine colic. The authors observed that most survey participants stated that the two main sources of information nominated by the participants as contributing to their knowledge were personal experiences (86%) and talking with veterinarians (73%).

Exchanging information with other owners was the main method of obtaining information for 3.97% of the respondents in this survey. In the context of disease recognition and prevention, the information obtained may refer to owners who share their experiences with individual cases. However, despite offering useful insights, it must be treated with care, as it does not always represent a reasoned approach. Internet consultations, cited by 3.31% of the respondents, are a vast source of information about diseases; however, it is necessary to exercise discernment to verify the credibility of the sources and avoid incorrect information. Equine owners’ knowledge of infectious diseases is influenced by many factors, including formal education, previous experience, access to information, ongoing awareness, regional culture, involvement of animal health professionals, and risk awareness. It is fundamental to promote education and continuous awareness among owners, seeking to increase their level of knowledge and, consequently, improve the health and well-being of horses.

## Conclusion

Infectious and parasitic diseases represent a crucial factor in decision making regarding the general management of horses in view of their significant impact on the health and well-being of animals and the sustainability of equestrian activities. The presence of these diseases can negatively affect the productivity, athletic performance, and reproduction of horses, in addition to having a considerable economic impact on the equestrian industry. To mitigate these adverse effects, it is imperative to implement effective control and prevention strategies based on solid principles of biosafety and epidemiological surveillance. In addition, education and awareness among owners, veterinarians, and professionals involved in equestrian activities are essential for the early identification of suspicious clinical signs and prompt reporting of cases. Investing in educational programs and increasing the awareness of equine owners in the state of Rio Grande do Norte about the main infectious diseases in horses is essential toward improving sanitary management and the general health of animals. Informed owners play an essential role in creating safer environments for their horses, thereby contributing to the sustainability of the equine industry.
